# Prevalence of Mental Health Problems During Virus Epidemics in the General Public, Health Care Workers and Survivors: A Rapid Review of the Evidence

**DOI:** 10.3389/fpubh.2020.560389

**Published:** 2020-11-11

**Authors:** Simeon Joel Zürcher, Philipp Kerksieck, Christine Adamus, Christian Markus Burr, Anja I. Lehmann, Flavia Katharina Huber, Dirk Richter

**Affiliations:** ^1^Center for Psychiatric Rehabilitation, University Hospital for Mental Health (UPD), Bern, Switzerland; ^2^University Hospital of Psychiatry and Psychotherapy, University of Bern, Bern, Switzerland; ^3^Public and Organizational Health, Epidemiology, Biostatistics and Prevention Institute, University of Zurich, Zurich, Switzerland; ^4^Department of Health Professions, Bern University of Applied Sciences, Bern, Switzerland

**Keywords:** COVID-19, epidemic, mental health problems, pandemic, prevalence, SARS-CoV-2

## Abstract

**Background:** The swift spread of SARS-CoV-2 provides a challenge worldwide. As a consequence of restrictive public health measures like isolation, quarantine, and community containment, the provision of mental health services is a major challenge. Evidence from past virus epidemics and the current SARS-CoV-2 outbreak indicate high prevalence rates of mental health problems (MHP) as short- and long-term consequences. However, a broader picture of MHP among different populations is still lacking.

**Methods:** We conducted a rapid review on MHP prevalence rates published since 2000, during and after epidemics, including the general public, health care workers, and survivors. Any quantitative articles reporting on MHP rates were included. Out of 2,855 articles screened, a total of 74 were included in this review.

**Results:** Most original studies on MHP were conducted in China in the context of SARS-CoV-1, and reported on anxiety, depression, post-traumatic stress symptoms/disorder, general psychiatric morbidity, and psychological symptoms. The MHP rates across studies, populations, and epidemics vary substantially. While some studies show high and persistent rates of MHP in populations directly affected by isolation, quarantine, threat of infection, infection, or life-threatening symptoms (e.g., health care workers), other studies report minor effects. Furthermore, even less affected populations (e.g., distant to epidemic epicenter, no contact history with suspected or confirmed cases) can show high rates of MHP.

**Discussion:** MHP vary largely across countries and risk-groups in reviewed studies. The results call attention to potentially high MHP during epidemics. Individuals affected directly by an epidemic might be at a higher risk of short or even long-term mental health impairments. This study delivers insights stemming from a wide range of psychiatric instruments and questionnaires. The results call for the use of validated and standardized instruments, reference norms, and pre-post measurements to better understand the magnitude of the MHP during and after the epidemics. Nevertheless, emerging MHP should be considered during epidemics including the provision of access to mental health care to mitigate potential mental impairments.

## Introduction

In the past two decades, many countries faced challenges in the realm of major infectious disease epidemics including SARS-CoV-1 (1), Swine flu (H1N1) (2), Middle East respiratory syndrome coronavirus (MERS-CoV) (3), avian influenza (H7N9) (4), Ebolavirus (5), and the recent worldwide SARS-CoV-2 outbreak (6). Epidemic outbreaks can result in high case fatality rates and morbidity (7, 8) and may require communities to introduce restrictive public health measures like isolation, mass quarantine, and community containment interventions in order to stop transmissions and save lives (9). In consequence, epidemics can cause a high individual and societal burden and can lead to substantial economic loss (7, 10–12). While considerable efforts rely on protective and treatment measures such as virus transmission pathways, clinical presentations, and the development of vaccinations, attention is only recently given to short or long-term mental health problems (MHP, hereafter defined as psychiatric/psychological symptoms and mental illness/disorders) (13) that may arise due to the different surrounding consequences of an epidemic in the general public, health care workers (HCW), and survivors of infectious diseases (survivors).

Epidemics can negatively impact a substantial part of the general public in many different ways such as feelings of a personal threat of being infected (7, 14, 15), worries about relatives and family members or losing loved ones (14–16), and protective measures like mass quarantining, the consequences of which leads to individual and social restrictions, and economic loss (14). As a result, these factors can elicit feelings of anxiety, anger, loneliness, grief, boredom and may lead to serious MHP (14, 15, 17). Furthermore, the extensive and sometimes controversial mass media coverage during epidemics may amplify uncertainty, loss of control and anxiety (14, 17). Aside from the general public, HCW are prone to different MHP since they usually face an immediate threat of infection through patient contact by working at the epidemic frontline. Studies suggest that HCW accounted for up to 57% of SARS-CoV-1, 27% of MERS-CoV, and 12% of Ebola cases in some countries, which frequently resulted in morbidity or even death (18, 19). In HCW, epidemics often result in difficult working conditions like staff shortage, increased workload (7), overwhelming patient numbers (7, 19), limited safety equipment (7), and quarantine or isolation after infectious disease transmission (7, 14). Furthermore, HCW often suffer social consequences like stigma (7, 20, 21), mistrust and violence (7) avoidance from relatives, and the fear of infecting others (21). Given the high risk of transmission, HCW often account for a substantial fraction of survivors, who frequently experience isolation, intensive treatment, stigmatization, and exposure to an immediate threat of morbidity or death (7, 22). To date, many studies exist that describe MHP related to epidemics across a wide range of populations. However, to the best of our knowledge no review covering MHP during epidemics currently exists.

### Objectives

The purpose of this rapid review is to provide an overview of MHP prevalence rates during and after large epidemics of the past two decades. This research is important for informing research and practice about potential mental health issues and implications that may arise in the context of the current SARS-CoV-2 epidemic. We aim to provide a broad picture of MHP that may arise across a wide range of populations including (a) the general public, (b) HCW, and (c) and virus disease survivors. To synthesizes and deliver context-sensitive knowledge, we used a rapid review approach. As compared to systematic reviews, rapid reviews are a form of systematic knowledge synthesis with accelerated review processes and streamlined methods aiming at providing relevant evidence in a timely and efficient manner (23).

## Materials and Methods

### Search Strategy

The rapid and dynamic development of the current situation with SARS-CoV-2 requires quick evidence synthesis in order to inform decision-making processes in health care systems. The methodology of this article is based on the practical guide for rapid reviews provided by WHO. The results described in this study reflect a descriptive synthesis of evidence. As common for rapid reviews, facilitated methods for search, selection and data extraction were used and no meta-analysis was performed (23). We undertook a review of evidence on prevalence rates during and after epidemic outbreaks on MHP in the general public, HCW, and survivors. The focus was on SARS-CoV-1, H1N1, MERS-CoV, H7N9, Ebolavirus, and SARS-CoV-2. PubMed was searched on April 1, 2020 with a broad search strategy (see Supplementary Table 1). These virus epidemics were included as we assume important parallels in the way they affect mental health. More specifically, they elicit a large degree of uncertainty, feelings of threat, and major consequences in social and work lives.

### Participants, Interventions, and Comparators

Any type of quantitative study that provided prevalence rates of MHP in adults (≥ 18 years) during and after epidemic outbreaks, published in English from the year 2000 to March 31, 2020 was included. Studies that measured MHP rates assessed by psychometrically validated instruments, diagnostic interview, and medical records (chart review), were also included. We excluded studies that used a qualitative design, that did not report on MHP prevalence rates (e.g., providing mean scores only), that did not provide prevalence rates based on previously defined cut-off values for a measurement instrument (e.g., median based sample splitting), and that included MHP measured by single questions/items. Studies on common seasonal influenza were also excluded. Furthermore, general states like social functioning, quality of life, generic fears (e.g., fear of contracting a virus or worries) or stigma were excluded.

Based on the titles and abstracts of studies, potential eligible studies of the database search were selected by one author (CA) using a co-developed standardized review form to assess study eligibility. Subsequently, one author (SJZ) assessed full texts for eligibility. Doubts and uncertainty in eligibility of a certain study were solved by discussion (SJZ and CA).

### Data Sources, Study Selection, and Data Extraction

An electronic data extraction form was developed to assess the characteristics of the included studies and the reported MHP prevalence rates. Data was extracted by four authors in parallel (SJZ, CA, PK, and FKH) and subsequently audited by another author. Collected items included: author(s), year of publication, country or region, number of participants, type of epidemic outbreak, time point of assessment, type of MHP assessed, MHP prevalence rate, and assessment method. Time point of assessment was coded as: during epidemic/hospital stay, post-epidemic/discharge including one-year follow-up ( ≤ 1y), between 1 and 4 years follow-up (>1-4y), or a combination of both if applicable (e.g., for longitudinal studies). MHP were categorized into anxiety, depression, post-traumatic symptoms/disorders (PTSD) or stress, burnout, psychiatric morbidity, and further MHP like hallucinations or insomnia. We used baseline assessment data for intervention studies that provided prevalence rates. Data was stratified by the following populations: (a) general public including general surveys, (b) HCW including all hospital staff, military duty members, and family members as caregivers involved in active treatment or in potential contact with patients, and (c) infectious disease survivors (that may include suspected cases in some studies). Data quality and strength of evidence was not rated in the current review. All authors who extracted data discussed possible uncertainties with the primary reviewer SJZ.

### Data Analysis

Included studies varied in assessment of MHP (e.g., questionnaires, diagnostic interviews), MHP instruments with applied cut-off scores, sampling methods and response rates, outbreak-related time points of assessments, and in regional differences in the magnitude/level of affect. Due to the approach chosen (rapid review), no meta-analysis was conducted. Therefore, a descriptive approach was utilized to synthesize reported MPH prevalence rates. If provided, we show MHP rates from a moderate degree of severity as defined by authors within original studies.

## Results

### Study Characteristics

**Figure 1:**
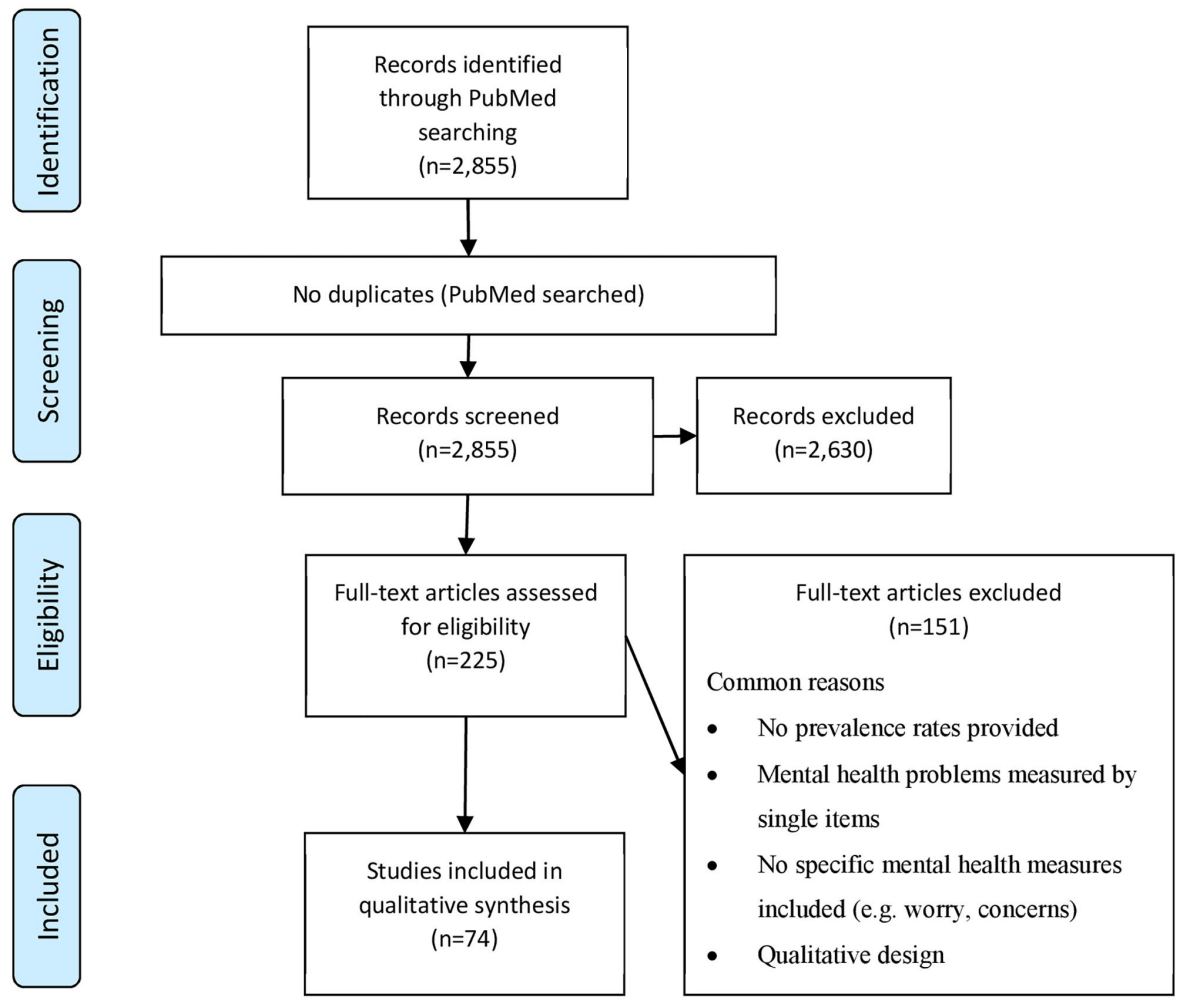
PRISMA flow diagram of the selection process of studies reported on mental health problem prevalence rates during or after virus epidemics retrieved for the rapid review.

Our PubMed search yielded 2,855 articles of which 2,630 articles were excluded based on title and abstract screening and 151 based on full-text screening. Most common reasons for exclusion during full-text screening were; (a) no prevalence rates provided (e.g., provision of mean scores for assessment instruments only), (b) mental health measured by single items (e.g., only one question used for assessment), (c) no specific mental health measures included (e.g., worries, concerns, quality of life), (d) qualitative design. Finally, 74 articles were included for the qualitative synthesis (see Figure 1). The majority of studies were cross-sectional in design and focused on MHP during SARS-CoV-1 (*n* = 41), followed by Ebolavirus (*n* = 12), MERS-CoV and SARS-CoV-2 (*n* = 7), H1N1 (*n* = 6), and H7N9 (n=1). About half of the studies in the general public used random sampling, while the majority of articles in HCW and survivors were non-random samples. The vast majority of studies was conducted in China, including Taiwan and Hong Kong (*n* = 39), followed by other countries in Asia (*n* = 14), in Africa (*n* = 12), and the American continent (mainly Canada; *n* = 6), with three studies conducted in Europe. We found *n* = 28, 26, and 20 studies that investigated the general public, HCW, and survivors, respectively. The vast majority of studies assessed MHP using self-reported questionnaires, while only few used standardized diagnostic interviews. Results stratified by general public, HCW, and survivors can be found in Tables 1–3.

**Table 1:** Reported prevalence rates with severity of mental health problems in the general public during and after epidemic outbreaks since 2000 in the respective countries/regions.

**References**	**Country/Region**	***N* (including other population)**	**Epidemic/Time point of assessment (during or post-epidemic)**	**Mental health problem^**a**^ (assessment instrument^**b**^): prevalence rates and severity if provided by original articles**
Cao et al. (24)	China	7,143	SARS-CoV-2/During	**Anxiety** (GAD-7): 2.7% moderate; 0.9% severe
Qiu et al. (25)	China, Hong Kong, Taiwan	52,730	SARS-CoV-2/During	**PTSD/Stress** (CPDI): 29.9% mild to moderate; 5.14% severe
Wang et al. (26)	China	1,210	SARS-CoV-2/During	**Anxiety** (DASS-21): 20.4% moderate; 8.4% severe/extremely severe
				**Depression** (DASS-21): 12.2% moderate; 4.3% severe/extremely severe
				**PTSD/Stress** (IES-R): 53.8% moderate/severe
				**PTSD/Stress** (DASS-21): 5.5% moderate; 2.6% severe/extremely severe
Wang et al. (27)	China	600	SARS-CoV-2/During	**Anxiety** (SAS): 0.67% moderate; 0% severe **Depression** (SDS): 2.5% moderate; 0.33% severe
Zhang and Ma (28)	China	263	SARS-CoV-2/During	**PTSD/Stress** (IES): 7.6% moderate/severe
Kamara et al. (29)	Sierra Leone	143	Ebolavirus/During	**Further MHP** (Med-rec): <1% alcohol/substance use disorder; 21% psychotic symptoms (e.g., hallucinations); 12% moderate to severe emotional disorder or depression
Koroma et al. (30)	Sierra Leone	10,011	Ebolavirus/During/Post ≤ 1y	**Further MHP** (Med-rec), any mental health disorders in various hospital types: <1% pre-Ebola; <1% during Ebola; ≤ 1% post-Ebola
Betancourt et al. (31)	Sierra Leone	1,008	Ebolavirus/Post ≤ 1y	**Anxiety** (HSCL-25): 1.3%
				**Depression** (HSCL-25): 1.4%
				**PTSD/Stress** (PSS-I): 11.3% likely PTSD
Jalloh et al. (32)	Sierra Leone	3,564	Ebolavirus/Post ≤ 1y	**Anxiety/Depression** (PHQ-4): 48.6% any symptoms **PTSD/Stress** (IES-R): 76.4% any symptoms; 27% levels of clinical concern for PTSD; 16% levels of probable PTSD diagnosis
Mollers et al. (33)	Netherlands	72	MERS-CoV/During	**PTSD/Stress** (IES-R): 22%
Al-Rabiaah et al. (34)	Saudi Arabia	174	MERS-CoV/During	**Anxiety** (GAD-7): 4.6% moderate; 0% severe
Jeong et al. (35)	Republic of Korea	1'692 (incl. HCW, Survivors)	MERS-CoV/Post ≤ 1y	***During isolation***: **Anxiety** (GAD-7): 47.2% MERS positive; 7.6% negative
				**Further MHP/Anger** (STAXI): 52.8% MERS positive; 16.6% negative
				***4-6 months after isolation***: **Anxiety** (GAD-7): 19.4% MERS positive; 3% negative
				**Further MHP/Anger** (STAXI): 30.6% MERS positive; 6.4% negative
Rubin et al. (36)	England, Scotland	997	H1N1/During	**Anxiety** (STAI-6): 23.8% symptoms; 2.1% high
Wang et al. (37)	China	419	H1N1/During	**Psychiatric morbidity** (SRQ-20): 8% quarantined group; 14% non-quarantined group
				**PTSD/Stress** (IES-R): 10.8% quarantined group; 16.9% non-quarantined group
Xu et al. (38)	China	1082	H1N1/During	**PTSD/Stress** (PCL-C): 2% symptomatic PTSD
Leung et al. (39)	Hong Kong	1,115	SARS-CoV-1/During	**Anxiety** (STAI): 12.6% quite/very anxious
Hawryluck et al. (40)	Canada	129 (incl. HCW)	SARS-CoV-1/During	**Depression** (CES-D): 31.2% **PTSD/Stress** (IES-R): 28.9%
Quah and Hin-Peng (41)	Singapore	1,202	SARS-CoV-1/During	**Anxiety** (CAS): 42.4% moderate; 2.9% high
Lau et al. (42)	Hong Kong	818	SARS-CoV-1/During	**PTSD/Stress** (IES): 13.3% males, 18.0% females moderate to severe; 1.3% males, 1.5% females severe
Lau et al. (43)	Hong Kong	818	SARS-CoV-1/During	**PTSD/Stress** (IES): 16% moderate to severe
Lee et al. (44)	Hong Kong	235	SARS-CoV-1/During	**Depression** (BDI): 12.3%
Chan et al. (45)	Hong Kong	122	SARS-CoV-1/During	**Anxiety** (STAI): 29.5% moderate; 4.1% high
Reynolds et al. (46)	Canada	1,057 (incl. HCW)	SARS-CoV-1/During	**PTSD/Stress** (IES-R): 14.6%
Sim et al. (47)	Singapore	415	SARS-CoV-1/During	**Psychiatric morbidity** (GHQ-28): 22.9%
				**PTSD/Stress** (IES-R): 25.8% high levels
Ko et al. (Ko et al., 28)	Taiwan	1,499	SARS-CoV-1/Post ≤ 1y	**Depression** (TDQ): 3.7% depressive symptoms
Lee et al. (48)	Hong Kong	146	SARS-CoV-1/Post ≤ 1y	**Depression** (CES-D): 32.4% elderly; 18.7% middle-aged
				**PTSD/Stress** (IES-R): 14.1% elderly; 4% middle-aged
Mihashi et al. (49)	China	187	SARS-CoV-1/Post ≤ 1y	**Psychiatric morbidity** (GHQ-30): 24.6% during the isolation period; 26.2% during the recovery period
Peng et al. (50)	Taiwan	1,278	SARS-CoV-1/Post ≤ 1y	**PTSD/Stress** (BSRS-5): 11.7%

a *PTSD, Post-traumatic stress disorder*.

b *BDI, Beck Depression Inventory; BSRS-5, 5-item Brief Symptom Rating Scale; CAS, B.A. Thyer's Clinical Anxiety Scale; CES-D, Center for Epidemiological Studies Depression Scale; CPDI, COVID-19 Peritraumatic Distress Index; DASS-21, Depression, Anxiety and Stress Scale; GAD-7, 7-item Generalized Anxiety Disorder Scale; GHQ, General Health Questionnaire; HSCL-25, Hopkins Symptom Checklist-25; IES-R: Impact of Event Scale–Revised; Med-rec, medical records; PCL-C, PTSD Checklist–Civilian Version; PHQ-4, Patient Health Questionnaire; PSS-I, PTSD Symptom Scale-Interview; SAS, Self-Rating Anxiety Scale; SDS, Self-Rating Depression Scale; SRQ-20, Self-Report Questionnaire; STAI, Spielberger State-Trait Anxiety Inventory; STAXI, State-Trait Anger Expression Inventory; TDQ, Taiwanese Depression Questionnaire*.

**Table 2:** Reported prevalence rates with severity of mental health problems in health care workers during and after epidemic outbreaks since 2000 in the respective countries/regions.

**References**	**Country/Region**	***N* (including other population)**	**Epidemic/Time point of assessment (during or post-epidemic)**	**Mental health problem^**a**^ (assessment instrument^**b**^): prevalence rates and severity if provided by original articles**
Lai et al. (51)	China	1,257	SARS-CoV-2/During	**Anxiety** (GAD-7): 32.3% mild; 7% moderate; 5.3% severe
				**Depression** (PHQ-9): 35.6% mild; 8.6% moderate; 4.9% severe
				**PTSD/Stress** (IES-R): 36.5% mild; 24.5% moderate; 10.5% severe
				**Further MHP/Insomnia** (ISI): 26.2% mild; 6.8% moderate; 1% severe
Sipos et al. (52)	Liberia	173	Ebolavirus/During	**Anxiety** (GAD-7): 2.3%
				**Depression** (PHQ-8): 2.3%
				**PTSD/Stress** (PCL): 4.0%
				**Further MHP/Insomnia** (ISI): 12.4%
Tang et al. (53)	China	102	H7N9/During	**PTSD/Stress** (PCL-C): 20.6%
Lee et al. (54)	Repuplic of Korea	359 during; 77 after	MERS-CoV/During	***During hospital shutdown***: **PTSD/Stress** (IES-R): 64.1% symptoms of; 51.5% diagnosis of PTSD
				***1 month after hospital shutdown (in those with PTSD diagnosis)*****:** **Anxiety** (HADS): 11%
				**Depression** (HADS): 15.1%
				**PTSD/Stress** (IES-R): 54.5% symptoms of; 40.3% diagnosis of PTSD
				**Psychiatric morbidity** (MINI): 5.5% major depression; 11% generalized anxiety disorder
Jung et al. (55)	Repuplic of Korea	147	MERS-CoV/Post ≤ 1y	**PTSD/Stress** (IES-R): 57.1% total; 32.0% moderate; 25.1% full PTSD
Mishra et al. (56)	India	271	H1N1/During	**Anxiety** (BAI): 1.5% moderate/high
Elizarraras-Rivas et al. (57)	Mexico	35	H1N1/Post ≤ 1y	**Anxiety** (DAQ): 71% moderate; 17% high
				**Depression** (CES-D): 34% low; 6% moderate; 3% high
				**PTSD/Stress** (PSS-10): 0% moderate; 3% high
Goulia et al. (58)	Greece	469	H1N1/Post ≤ 1y	**Psychiatric morbidity/stress** (GHQ-28): 20.7% mild/moderate; 6.8% severe
Bai et al. (20)	Taiwan	338	SARS-CoV-1/During	**PTSD/Stress** (DSM-IV): 5% acute stress disorder
Chan and Huak (59)	Singapore	661	SARS-CoV-1/During	**Psychiatric morbidity** (GHQ-28): 27%
				**PTSD/Stress** (IES): 20%
Chong et al. (60)	Taiwan	1257	SARS-CoV-1/During	**Psychiatric morbidity** (GHQ-12): 75.3%
Nickell et al. (61)	Canada	2,001	SARS-CoV-1/During	**Psychiatric morbidity** (GHQ-12): 29%
Verma et al. (21).	Singapore	1,050	SARS-CoV-1/During	**Psychiatric morbidity** (GHQ-28): 14.1% of general practitioners; 6% TCM practitioners
Chen et al. (62)	Taiwan	131	SARS-CoV-1/During	**PTSD/Stress** (IES): 11% total; 17% in high-risk units; 10% in low risk units
Lu et al. (63)	Taiwan	127	SARS-CoV-1/During	**Psychiatric morbidity** (GHQ-12): 17.3%
Su et al. (64)	Taiwan	102	SARS-CoV-1/During	**Depression** (BDI): 27.5% total; 38.5% in SARS units; 6.7% in non-SARS units
				**PTSD/Stress** (DTS-C): 33% SARS units; 19% non-SARS units
				**Further MHP/Insomnia** (PSQI): 37.1% SARS units; 9.4% non-SARS units
Tam et al. (65)	Hong Kong	652	SARS-CoV-1/During/Post ≤ 1y	**Psychiatric morbidity** (GHQ-12): 56.7%
Lung et al. (66)	Taiwan	During 127 Follow-up 123	SARS-CoV-1/During/Post ≤ 1y	***During epidemic***: **Psychiatric morbidity** (CHQ-12): 17.3%
				***1-year follow-up***: **Psychiatric morbidity** (CHQ-12): 15.4%
Sim et al. (67).	Singapore	277	SARS-CoV-1/Post ≤ 1y	**Psychiatric morbidity** (GHQ-28): 20.6% **PTSD/Stress** (IES-R): 9.4%
Phua et al. (68)	Singapore	96	SARS-CoV-1/Post ≤ 1y	**Psychiatric morbidity** (GHQ-28): 18.8%
				**PTSD/Stress** (IES): 17.7%
Lin et al. (69)	Taiwan	92	SARS-CoV-1/Post ≤ 1y	**PTSD/Stress** (DTS-C): 19.3% likely PTSD
				**Psychiatric morbidity** (GHQ-12): 47.8%
Lancee et al. (70)	Canada	133	SARS-CoV-1/Post ≤ 1y	**Depression** (SCID): 3.8% major depression
				**Stress/PTSD** (SCID): 1.5%
				**Further MHP** (SCID): 0.8% panic disorder; 1.5% substance abuse/dependence; 6.8% any new axis I diagnosis
Maunder et al. (71)	Canada	587 exposed; 182 non exposed	SARS-CoV-1/Post >1-4y	**PTSD/Stress** (IES): 13.8% high in exposed; 8.4% high in non-exposed group
				**PTSD/Stress** (K10): 44.9% high in exposed; 30.2% high in non-exposed
				**Burnout** (MBI-EE): 30.4% high in exposed; 19.2% high in non-exposed
Wu et al. (72)	China	549	SARS-CoV-1/Post >1-4y	**Depression** (CES-D): 22.8%
				**PTSD/Stress** (IES-R): 10.1% high PTSD symptoms
				**Further MHP/Alcohol-related symptoms** (NHSDA-adapted): 19%
Wu et al. (73)	China	549	SARS-CoV-1/Post >1-4y	**PTSD/Stress** (IES-R): 10% high level at any time during follow-up period; 4% still had high level at 3-year follow-up
Liu et al. (74)	China	549	SARS-CoV-1/Post >1-4y	**Depression** (CES-D): 14% moderate; 8.8% high level
				**PTSD/Stress** (IES-R): 10% high level

a *PTSD, Post-traumatic stress disorder*.

b *BAI, Beck Anxiety Inventory; BDI, Beck Depression Inventory; CES-D, The Center for Epidemiologic Studies Depression Scale; CHQ-12, Chinese Health Questionnaire; DAQ, Death Anxiety Questionnaire; DSM-IV, Diagnostic and Statistical Manual of Mental Disorders−4th edition; DTS-C, Davidson Trauma Scale—Chinese version; GAD-7, 7-item Generalized Anxiety Disorder Scale; GHQ, General Health Questionnaire; HADS, Hospital Anxiety and Depression Scale; IES-R, Impact of Event Scale—Revised; ISI, Insomnia Severity index; K10, Kessler Psychological Distress Scale; MBI-EE, Maslach Burnout Inventory—Emotional Exhaustion Scale; MINI, Mini International Neuropsychiatric Interview; NHSDA-adapted, 7-items adaptation of the National Household Survey on Drug Abuse; PCL, PTSD Checklist; PCL-C, PTSD Checklist—Cvilian Version; PHQ, Patient Health Questionnaire; PSQI, Pitssburgh Sleep Quality Index; PSS-10, Perceived Stress Scale; SCID, Structured Clinical Interview for DSM-IV*.

**Table 3:** Reported prevalence rates with severity of mental health problems in survivors during and after epidemic outbreaks since 2000 in the respective countries/regions.

**References**	**Country/Region**	***N* (including other population)**	**Epidemic/Time point of assessment (during or post-epidemic)**	**Mental health problem^**a**^ (Assessment Instrument^**b**^): Prevalence rates and severity if provided by original articles**
Bo et al. (75)	China	714	SARS-CoV-2/During	**PTSD/Stress** (PCL-C): 96.2% significant symptoms
Ji et al. (76)	Sierra Leone	18	Ebolavirus/During	**Anxiety** (SCL-90-R): 94.4% phobic anxiety; 83.3% anxiety
				**Further MHP** (SCL-90-R): 83.3% obsession-compulsion; 94.4% hostility; 72.2% paranoid ideation
Howlett et al. (77)	Sierra Leone	35	Ebolavirus/During	**Anxiety** (MINI-plus/MMSE): 27.5% anxiety symptoms
				**Depression** (MINI-plus/MMSE): 30% depressive symptoms
				**Further MHP/Insomnia** (MINI-plus/MMSE): 52.5%
Etard et al. (78)	Guinea	713	Ebolavirus/Post ≤ 1y	**Depression** (CES-D): 17%
Guetiya Wadoum et al. (79)	Sierra Leone	246	Ebolavirus/Post ≤ 1y	**Further MHP** (ESMHCMAF): 3.3% hallucinations; 24.4% psychotrauma; 10.1% insomnia
Keita et al. (80)	Guinea	256	Ebolavirus/Post ≤ 1y	**Depression** (CES-D/ICD-10): 15%; 10.9%
				**PTSD** (ICD-10): 1.2%
Pers et al. (81)	Guinea	142	Ebolavirus/Post >1-4y	**Depression** (CES-D): 18.3%
de St Maurice et al. (82)	Liberia	329	Ebolavirus/Post ≤ 1-4y	**Anxiety** (Med-rec): 13%
				**Depression** (Med-rec): 13%
				**Insomnia** (Med-rec): 15%
Kim et al. (83)	Republic of Korea	27	MERS-CoV/During	**Depression** (PHQ-9): 40.7%
Lee et al. (84)	Republic of Korea	72	MERS-CoV/Post >1-4y	***12 months follow-up***: **Depression** (PHQ-9): 26.9%
				**PTSD/Stress** (IES-R): 42.3%
				**Further MHP/Fatigue** (FSS): 48.1% ***18 months follow-up***: **Depression** (PHQ-9): 17.3%
				**PTSD/Stress** (IES-R): 26.9%
				**Further MHP/Fatigue** (FSS): 32.7%
Cheng et al. (85)	Hong Kong	180 (incl. HCW)	SARS-CoV-1/Post ≤ 1y	**Anxiety** (BAI): 23.4% mild/moderate; 24.6% moderate/severe; 7.3% severe
				**Depression** (BDI): 24.7% mild/moderate; 19.1% moderate/severe; 6.7% severe
Sheng et al. (86)	Hong Kong	102 (incl. HCW)	SARS-CoV-1/Post ≤ 1y	***Acute phase***: **Further MHP** (NPSC, examples): 46.1% insomnia; 36.3% low mood; 2% suicidal idea; 26.5% fear and panic; 36.3% tension; 5.9% hallucinations
				***Convalescent phase***: **Psychiatric morbidity** (GHQ-28): 64.7% **Further MHP** (NPSC, examples): 22.5% insomnia; 18.6% low mood; 0% suicidal idea; 13.7% fear and panic; 20.6% tension; 1% hallucinations
Wu et al. (87)	Hong Kong	131 (incl. HCW)	SARS-CoV-1/Post ≤ 1y	***1 month follow-up*****:** **Anxiety** (HADS): 13%
				**Depression** (HADS): 18% **PTSD/Stress** (IES-R): 4% all subscales; 12% intrusion; 9% avoidance; 15% hyperarousal ***3 months follow-up*****:** **Anxiety** (HADS): 14%
				**Depression** (HADS): 13%
				**PTSD/Stress** (IES-R): 5% all subscales; 10% intrusion; 8% avoidance; 9% hyperarousal
Wu et al. (88)	Hong Kong	195	SARS-CoV-1/Post ≤ 1y	**Anxiety** (HADS): 14%
				**Depression** (HADS): 18%
				**PTSD/Stress** (IES-R): 6%
Kwek et al. (89)	Singapore	63 (incl. HCW)	SARS-CoV-1/Post ≤ 1y	**Anxiety** (HADS): 17.5% at least moderate anxiety
				**Depression** (HADS): 11.1% at least moderate depression
				**PTSD/Stress** (IES): 41.7% at least moderate; 36.7% at least severe
Lee et al. (90)	Hong Kong	96 (incl. HCW)	SARS-CoV-1/Post ≤ 1y	**Anxiety** (DASS-21): 36.7% moderate/severe; 14.4% extremely severe
				**Depression** (DASS-21): 36.3% moderate/severe; 4.4% extremely severe
				**Psychiatric morbidity** (GHQ-12): 64% total; 90.3% HCW; 49.1% non-HCW
				**PTSD/Stress** (IES-R): at least moderate level on subscales: 32.2% Intrusion; 20.0% avoidance; 22.2% hyperarousal
Hong et al. (91)	China	70	SARS-CoV-1/Post ≤ 1y and >1-4y	**PTSD/Stress** (DSM-IV): 44.1% met criteria in at least one follow-up visit
Lam et al. (92)	Hong Kong	181 (incl. HCW)	SARS-CoV-1/Post >1-4y	**Depression** (HADS/SCID): 35.6%; 39%
				**PTSD/Stress** (IES-R): 27.9% intrusion; 17.6% avoidance; 33.5% hyperarousal
				**Further MHP** (SCID): 42.5% at least one active psychiatric illness; 54.5% PTSD; 36.4% somatoform pain disorder; 32.5% panic disorder; 15.6% obsessive compulsive disorder
				**Fatigue** (CFQ/CFS): 40.3%; 27.1%
Mak et al. (11)	Hong Kong	90 (incl. HCW)	SARS-CoV-1/Post >1-4y	***Since outbreak***: **PTSD/Stress** (IES-R/SCID): 47.8%
				**Further MHP** (SCID): 58.9% any diagnosis; 46.7% depressive disorder; 21.1% anxiety disorders
				***30 months post-SARS***: **Anxiety** (HADS): 15.6% moderate/severe anxiety
				**Depression** (HADS): 18.9% moderate/severe depression
				**PTSD/Stress** (IES-R/SCID): 25.6%
				**Further MHP** (SCID): 33.3% any diagnosis; 15.6% depressive disorder; 14.6% anxiety disorders
Mak et al. (93)	Hong Kong	90 (incl. HCW)	SARS-CoV-1/Post >1-4y	**PTSD** (SCID): total of 47.8% at some time point after the SARS outbreak; 25.6% at 30 months post-SARS

a *PTSD, Post-traumatic stress disorder*.

b *BAI, Beck Anxiety Inventory; BDI, Beck Depression Inventory; CES-D, The Center for Epidemiologic Studies Depression Scale; CFQ, Chalder Fatigue Questionnaire; CFS, modified criteria for chronic fatigue syndrome (CFS) according to the Centers for Disease Control and Prevention; DASS-21, Depression, Anxiety and Stress Scale; DSM-IV, Diagnostic and Statistical Manual of Mental Disorders−4th edition; ESMHCMAF, Ebola Survivors Mobile Health Clinic Medical Assessment Form; FSS, Fatigue Severity Scale; GHQ, General Health Questionnaire; HADS, Hospital Anxiety and Depression Scale; ICD-10, International Classification of Diseases−10th edition; IES-R, Impact of Event Scale—Revised; Med-rec, medical records; MINI-plus, Mini International Neuropsychiatric Interview; MMSE, Mini Mental State Examination; NPSC, Neuropsychiatric Symptoms Checklist; PCL-C, PTSD Checklist—Cvilian Version; PHQ-9, Patient Health Questionnaire; SCID, Structured Clinical Interview for DSM-IV; SCL-90-R, Symptom Checklist*.

### Synthesized Findings

#### General Public

Range of prevalence rates across original articles were as follows: anxiety (0.7–47.2%), depression (1.4–32.4%), any anxiety/depression symptoms combined (48.6%), PTSD/stress (2.0–76.4%), and psychiatric morbidity (8.0–26.2%). The rates of further MHP included any mental disorder (<1.0%), alcohol/substance use disorders (<1.0%), anger (6.4–52.8%), moderate to severe emotional disorder or depression (12.0%), and psychotic symptoms like hallucinations (21.0%). The highest and lowest rates of anxiety were found in MERS-CoV (48.6%), and SARS-CoV-2 (0.7%), respectively. For depression the highest rates were found in SARS-CoV-1 (32.4%) and the lowest in Ebolavirus (1.4%). For PTSD/stress, the highest rates were shown for Ebolavirus (76.4%), and the lowest in H1N1 (2.0%). Psychiatric morbidity was highest in SARS-CoV-1 (26.2%) and lowest in H1N1 (8.0%). The majority of studies in the general public reported on MHP during or shortly after ( ≤ 1y) epidemic outbreaks. To the best of our knowledge there are no studies published reporting on potential late sequela >1y after an epidemic.

#### Health Care Workers

Range of prevalence rates were as follows: anxiety (1.5–88.0%), depression (2.3–49.1%), PTSD/stress (1.5–71.5%), burnout (19.2–30.4%), and psychiatric morbidity (6.0–75.3%). The rates of further MPH included any new Axis 1 diagnosis (6.8%), insomnia (9.4–37.1%), and substance abuse or alcohol related symptoms (1.5–19.0%). The full range of rates in anxiety were both found in H1N1 (1.5–88.0%). For depression, the highest rates were found in SARS-CoV-2 (49.1%) and the lowest in Ebolavirus (2.3%). For PTSD/stress, the highest rates were shown for SARS-CoV-2 (71.5%) and the lowest for SARS-CoV-1 (1.5%). Highest and lowest rates for psychiatric morbidity were both found for SARS-CoV-1 (6.0–75.3%). The majority of studies in HCW reported on MHP during or shortly after ( ≤ 1y) epidemic outbreaks. Four studies reported on MHP with follow-up assessments of up to 4 years in the context of SARS-CoV-1. MHP differed substantially even when separated by follow-up time points. Results show that rates can still be high at follow-up time points >1y (e.g., Burnout rates of 30.4%).

#### Survivors

Range of prevalence rates were as follows: anxiety (13.0–94.4%), depression (11.0–50.5%), PTSD/stress (1.2–96.2%), and psychiatric morbidity (49.1–90.3%). Furthermore, the rates of further MHP included any psychiatric diagnosis (33.3–58.9%), fatigue (27.1–48.1%), fear and panic (13.7–26.5%), hallucinations (1–5.9%), insomnia (10.1–52.5%), low mood (18.6–36.3%). obsession-compulsion (15.6–83.3%), panic disorder (32.5%), paranoid ideation (72.2%), somatoform pain disorder (36.4%), suicidal ideation (2.0%), and tensions/hostility (20.6–94.4%). The highest and lowest rates of anxiety were fund in Ebolavirus (94.4%), and SARS-CoV-1/Ebolavirus (13%), respectively. Depression was highest in SARS-CoV-1 (50.5%) and lowest in ebolavirus (11%). For PTSD/stress, the highest rates were shown for SARS-CoV-2 (96.2%) and lowest for Ebolavirus (1.2%). Psychiatric morbidity was described only in SARS-CoV-1 (49.1–90.3%). As in HCW, studies in survivors across different follow-up time points show a broad range of MHP rates. Studies including assessments >1y post-epidemic show that rates can still be high.

## Discussion

### Summary of Main Findings

This rapid review presents a descriptive synthesis of 74 original articles using streamlined review methodology in order to provide a broad overview of MHP in a timely manner. We found a wide range of MHP including anxiety, depression, PTSD and stress related symptoms or disorders, psychiatric morbidity, and many further MHP like paranoid ideation, hallucinations, and insomnia that may occur in the general public, HCW or survivors during and after epidemic outbreaks. Original articles commonly describe simple prevalence rates rather than reporting changes in MHP since epidemic outbreaks. Aside from methodological issues and the large heterogeneity of original studies (e.g., poor validation, different cut-offs for case definition), which makes it difficult to understand the magnitude of the problem, MHP can be more prevalent in all three populations in the context of an epidemic. These problems may be substantial and can persist over time in HCW and survivors more directly affected by the epidemic threat. However, it should be noted that epidemic circumstances can also yield positive impacts on mental health like spending more time on physical activity and taking more care of one's mental health (43).

### General Public

MHP ranged widely both across the general public and in all epidemics, which makes it difficult to estimate the magnitude and associated characteristics that may aggravate MHP. However, many studies investigated risk and protective factors of MHP. Although some controversy exists among studies, a higher level of epidemic exposure (e.g., living proximity to epidemic epicenter, contact history to high prevalent virus regions) (48, 94), hospitalization during epidemic (47), being quarantined (95), or having infected family members (24, 38, 44) may aggravate MHP. Further risk factors include being female (37, 38, 90, 94), chronic physical illness (85), poor self-rated health (26), and dissatisfaction with measures controlling the virus (37). Furthermore, many studies reported problems like loneliness, boredom, anger, worries about family members (26), and financial problems or economic loss (3, 42, 56, 96) that negatively interfere with mental health. In contrast, accurate health information (e.g., treatment, local outbreak situation) (26), particular precautionary measures (e.g., hand hygiene, wearing a mask) (26), social support (24, 43, 95), and appraisals and coping strategies (15, 85) may be protective.

### Health Care Workers and Survivors

Similarly, HCW and survivors showed a wide range of mental health impacts. However, MHP rates in these populations may be more substantial than in the general public. HCW that were directly involved in patient care (21), working in high risk units and with infected patients (62, 64, 71, 97) conscripted workers (62), or that underwent quarantine during outbreak (20, 74) were found to be associated with a higher risk of MHP. Furthermore, younger age (21, 64), being single (59, 74), fear of adversely affecting relatives (97, 98), pre-exposure to traumatic events or history of MHP (64, 70, 74) were also found to be associated with a higher risk of MHP. In contrast, adequate professional education and training (53, 70, 71), support from colleagues (59), appropriate information and communication (directives, precautionary measures, disease information) (59), and altruistic risk acceptance (74) were found to be protective. In survivors MHP may be aggravated by a history of mental illness (35), the fear of permanent damage or death (85, 88), longer duration of quarantine (40), having physical late sequelae (81), and impairment of ability to work (92). Furthermore, survivors that are HCW were shown to be more susceptible to long term MHP compared to non-HCW survivors (90, 99).

### Mental Health Problems and Methodological Issues

The methodological characteristics and quality of studies in assessing MHP ranges widely. We found only few studies that did not utilize a cross-sectional design without repetition. Further, most cross-sectional studies did not report any comparative data from which the change of prevalence rates due to the epidemic could be estimated. Sampling characteristics were also varying. Only about half of the studies in the general public were based on representative samples. As many studies were conducted during or shortly after the peak phase of the epidemic, results have to be regarded as acute stress reactions that do not allow for inference of longer-lasting MHP. While some authors used well-established and widely used instruments and standardized diagnostic interviews [e.g., Ji et al. (76) or Lancee et al. (70)], others used instruments with unclear quality [e.g., Guetiya Wadoum et al. (79)]. Besides the possibility of biased results, this approach makes it challenging to identify clinically relevant cases. With respect to the application of diagnostic instruments, cut-off values might vary between countries and cultures. Therefore, a lack of validated, country-specific, cut-off values of the measurement instruments might be problematic (32).

### Future Directives and Implications for Research, Policy, and Practice

#### Monitoring MHP as a Tool for Mental Health Care Provision

As shown by this review, MHP may be prevalent across a broad range of populations. In this vein, clinical monitoring of risk groups that are vulnerable to psychological impairments due to the current SARS-CoV-2 epidemic is essential (100). Pfefferbaum and North (100) pointed out, that the monitoring of psychosocial needs should assess SARS-CoV-2–related stressors, secondary adversities, psychosocial effects, and indicators of vulnerability. Besides others, routine outcome monitoring (101) as a measurement feedback system, apps for (self-)monitoring of mood, sleep-quality, or medication adherence (102), and artificial intelligence predicting relevant psychiatric outcomes (103), are available for public mental health monitoring. In the best case, mental health service providers should be aided by e-monitoring during epidemics. As mentioned above, in research MHP should be assessed by standardized diagnostic interviews or measurement instruments, enabling appropriate case detection identifying risk groups in order to inform policy and practice. For profound and substantial planning of the mental health infrastructure, MHP associated with SARS-CoV-2 need to be identified regarding potential evolving short or long term treatments.

#### Access to Mental Health Service in Epidemics

Furthermore, access to mental health services for those in need is paramount during the SARS-CoV-2 crisis, especially when social isolation is experienced (104). Beside the psychosocial consequences of public health measures such as quarantine (14), acute viral infection is unknown but likely to be accompanied by substantial neuropsychiatric symptoms (anxiety, depression, and trauma-related symptoms) as a host immunologic response to the infection (22). Mental health care interventions are expected to reduce symptoms such as PTSD (105). However, during epidemic scenarios care needs to be adapted to upcoming circumstances by respective governments in order to prevent or support individuals with MHP (106). In epidemic conditions, where consultation in-person is restricted there are important implications for digital health approaches. Online psychotherapy and consultation might help to improve access to mental health care, particularly in times of quarantine and isolation (107, 108). It does need to be highlighted that the effectiveness of online services for the improvement of mental health services requires further assessment (109). Consequently, the outbreak of SARS-CoV-2 calls for rapid reports and insights, as well as long-term health service research focusing on both remote and in-person mental health resources during epidemics (110, 111).

#### Implications for HCW as a Highly Demanded Group

Working conditions play an important role in mental health. For HCW, protective working conditions such as social support, constructive communication and staff training, and education have already been mentioned in some studies (53, 70, 71). Employers should consider strengthening these resources by implementing support systems and coping management strategies. Besides such protective factors there might be even health promoting occupational aspects to be considered. For HCW, the intent to help can buffer mental health-impairing consequences (74) but might be a rewarding factor in and of itself (112). It is also conceivable that enhanced public attention can trigger public appreciation of HCW. Furthermore, HCW could move to the political fore promoting improvements in the working conditions. Such rewarding aspects should be investigated in future studies.

#### Implications for the General Public

The importance of social support for mental health has been highlighted by several studies (24, 43, 95). Digital communication with friends, relatives and colleagues might buffer the negative effects of loneliness and separation. Although most of the studies have highlighted stressors and protective factors to cope with these stressors, there might even be rewarding aspects in times of an epidemic. Some positive mental health-related factors like family support, mental health awareness and lifestyle changes such as time to rest, to relax or to exercise have already been investigated (43). During epidemics, a substantial proportion of individuals might be confronted with altered working conditions like teleworking, which is generally associated with pros and cons for mental health (113). Future studies should examine ways to reduce the negative impact of home-office situations in times of an epidemic crisis.

#### Information Policies for Public Crisis Management

Many studies have highlighted the role of timely and adequate information that should be provided (26). Epidemics with escalating case numbers and mass quarantine convey the impression of a serious personal threat and increase feelings of anxiety, loss of control and being trapped (114). The extensive mass and social media coverage is associated with public concerns and may contribute to negative psychological effects (75, 115). Appropriate information and education programs may not only help to decrease anxiety (45) but also benefit in adopting protective measures (116). Thus, adequate media is essential for the promotion of protective measures (115). Besides the responsibility of (health-) authorities to provide adequate information, it is necessary to understand the development of public attitudes to better target communication strategies, particularly with the rise of fake news and conspiracy theories (117). Furthermore, strengthening health literacy (118) appears to be important in enabling people to evaluate the relevant information. Consequently, it is of advantage to inform individuals that mild stress reactions may occur in such an epidemic that are not necessarily clinically relevant. However, a diagnostic clarification must be provided if justified by psychological strain. Generally, the application of health behavior theories in research of public attitudes and behaviors would enhance the development of public health interventions that address the mental health-impairing processes of an epidemic crisis.

#### Addressing the Needs of Subpopulations in Public Health Policy

With regard to the general public, the consideration of subpopulations was mainly neglected. For instance, people with mental illness (119) or children and families that might be victims of domestic violence, particularly in times of quarantine (120). Also, for the elderly, the effects of social distancing could lead to isolation, loneliness and severe mental health consequences (121). It is generally accepted to assume that people lacking resources (such as financial, cultural or social resources) might be more vulnerable within a crisis (122). Given this, future studies should examine mental-health effects for specific subpopulations. This would result in targeted interventions in these populations in addition to general public mental health approaches.

### Strengths and Limitations

An important strength of our study is the inclusion of a broad range of populations that may be affected by MHP during or after an epidemic. This rapid review provides an essential overview of a highly relevant public health topic since the impact of impaired mental health itself on individuals, society and economy can be substantial. Furthermore, the data shown (Tables 1–3) allows for further interpretations and delivers insights to aspects that are of interest for researchers, practitioners and policy planning (e.g., country specific prevalence rates). Limitations may arise from the methods used to screen and extract the evidence for this article. To provide evidence in a timely manner, a rapid review is the method of choice as information need is immediate. This rapid review differs from a systematic review in several aspects. First, we focused our search strategy on PubMed and did not additionally screen reference lists of relevant articles. Second, the steps of screening and eligibility of research articles were performed by one author, respectively. Third, data was not extracted independently by two authors but were mutually controlled after extraction. Additionally, no quality assessment of the studies was conducted. Further limitations arise from the large heterogeneity and methodological issues (see section Mental Health Problems and Methodological Issues). At the same time, the heterogeneity of integrated studies is an asset, as they offer an extensive perspective on the studied issue.

## Conclusion

In this rapid review of 74 original articles, we found a large range in prevalence rates of MHP such as anxiety, depression, post-traumatic stress symptoms or disorders, during and after epidemics across the general public, HCW, and survivors. MHP might be especially prominent among HCW and survivors that are directly affected by epidemics and face a real threat of infection and difficult circumstances like isolation/quarantine or difficult working conditions. As shown by various original studies, MHP across all populations can be substantially influenced by risk and protective factors, some of which are modifiable like social support and appropriate information by authorities. From a clinical point of view, policy makers and health care providers should be aware of potential short term or even persistent MHP. This is particularly relevant in planning of mental health infrastructure at large scale to encounter MHP elicited due to SARS-CoV-2 epidemic. Interventions should therefore rely on a comprehensive assessment combining risk factors for and symptoms of MHP considering their potential short or long term persistence. Short term MHP like stress reactions can generally be expected under the circumstances of an epidemic and should be distinguished from long term consequences or mental illness. Consequently, it may be required to develop and disseminate psychiatric programs based on the specific characteristics of the SARS-CoV-2 epidemic by integrating early diagnosis of determinants that anticipate a short or long-term course of treatment. During epidemics, mental health care needs to be adapted to changing circumstances in order to grant access and treatment to those in need. Digital mental health approaches can support access to care for the public. This allows for psychological monitoring and treatment when in-person consultations are not possible. Yet, digital health interventions are still in developmental stages and need further assessment. During lockdowns, they seem to be a relevant supplement to the provision of in-person mental health care. Furthermore, HCW that often account for a substantial fraction of virus cases need to be supported. However, health authorities and policy makers should keep in mind separating short-term acute stress reactions from long-term mental illness.

It is of note that many original studies used different approaches and show methodological diversity in the assessment of MHP, which at least partly explains the broad range of MHP. Thus, results should be treated with some caution since a comparison of prevalence rates across studies and assessment of magnitude of MHP is currently not possible. Future studies should monitor MHP with standardized methods and apply comparisons with country-specific norms and provide changes in prevalence rates in order to gain a better understanding of MHP, to learn about influential factors, and how to provide appropriate access to mental health care during epidemics. Although, this was out of scope for this review, evidence of MHP in vulnerable populations such as children, the elderly especially when socially isolated or people with pre-existing mental illness seems to be scarce and should be covered in future studies.

## Data Availability Statement

The original contributions presented in the study are included in the article/supplementary materials, further inquiries can be directed to the corresponding author/s.

## Author Contributions

DR and SJZ contributed to the design of the study, data acquisition, data interpretation, manuscript development, and revisions. PK contributed to data acquisition, data interpretation, manuscript development and revisions. CA and FKH contributed to data acquisition and manuscript revisions. CB contributed to data interpretation and manuscript revision. AIL contributed to data interpretation, manuscript development and revisions. All authors approved the final version of the submitted manuscript.

## Conflict of Interest

The reviewer AV declared a shared affiliation, with no collaboration, with several of the authors, PK and AIL, to the handling editor at the time of review. The remaining authors declare that the research was conducted in the absence of any commercial or financial relationships that could be construed as a potential conflict of interest.
