# Supplementary material for: Prevalence of Mental Health Problems During Virus Epidemics in the General Public, Health Care Workers and Survivors: A Rapid Review of the Evidence
Source: Front Public Health. 2020 Nov 11;8:560389. doi: 10.3389/fpubh.2020.560389 (PMC7688009; doi:10.3389/fpubh.2020.560389)
Supplement: Supplementary file 1 [file Table_1.docx]

# Supplements

**Supplementary Table 1** PubMed search strategy (search at April 1, 2020)

| Population | Sars*[Title/Abstract] OR COVID*[Title/Abstract] OR 2019-nCoV[Title/Abstract] OR Corona[Title/Abstract] OR Coronavirus[Title/Abstract] OR Mers*[Title/Abstract] OR “Severe Acute Respiratory”[Title/Abstract] OR Influenza*[Title/Abstract] OR Flu[Title/Abstract] OR Grippe[Title/Abstract] OR Ebola*[Title/Abstract] |
| --- | --- |
| AND |  |
| Outcomes | Mental[Title/Abstract] OR Psychological[Title/Abstract] OR depress*[Title/Abstract] OR schizophren* OR psychosis OR psychotic OR anxiety OR bipolar OR Stress[Title/Abstract] OR PTSD [Title/Abstract] OR Emotional [Title/Abstract] OR Trauma*[Title/Abstract] |
| NOT |  |
| Conditions / Interventions | Coronary[Title] OR Cardiac[Title] OR Cardiovascular[Title] OR “Physical activity”[Title] OR Biomarkers[Title] OR Injury[Title] OR Anaesthesia[Title] OR Melatonin[Title] OR Validation[Title] OR Pain[Title] OR Obesity[Title] OR Opioid[Title] OR fentanyl[Title] OR Orthopaedic[Title] OR Vaccin*[Title]) |
